# Dynamic differentiation of F4/80+ tumor-associated macrophage and its role in tumor vascularization in a syngeneic mouse model of colorectal liver metastasis

**DOI:** 10.1038/s41419-023-05626-1

**Published:** 2023-02-13

**Authors:** Ting Qiao, Wanli Yang, Xiangchuan He, Ping Song, Xiao Chen, Ruijie Liu, Jian Xiao, Xiaoli Yang, Mingqi Li, Yudan Gao, Guoan Chen, Yi Lu, Jian Zhang, Jing Leng, Huan Ren

**Affiliations:** 1 School of Medicine, Southern University of Science and Technology, 518055 Shenzhen, China; 2 Department of Immunology, Harbin Medical University, Harbin, China; 3 Chongming Hospital affiliated to Shanghai University of Medicine and Health Sciences, Shanghai, China; 4 Clinical Center for BioTherapy & Institutes of Biomedical Sciences, Zhongshan Hospital, Fudan University, Shanghai, China; 5 Department of Ophthalmology, Jiarun Hospital of Harbin, Harbin, China; 6 Department of Microbiology & Immunology, Guangxi Chinese Medicine University, Nanning, China; 7 Guangxi Key Laboratory of Translational Medicine for Treating High-incidence Infectious Diseases with Integrative Medicine, Nanning, China; 8 Department of Colorectal Surgery, the 3rd Hospital Affiliated to Harbin Medical University, Harbin, China; 9 Guangdong Provincial Key Laboratory of Cell Microenvironment and Disease Research, Shenzhen, Guangdong China

**Keywords:** Cancer microenvironment, Tumour angiogenesis, Metastasis, Kupffer cells

## Abstract

Tumor-associated macrophages (TAMs) are highly heterogeneous and play vital roles in tumor progression. Here we adopted a C57BL/6 mouse model imitating the late-stage colorectal liver metastasis (CRLM) by Mc38 colorectal cancer cell injection via the portal vein. With serial sections of CRLM biopsies, we defined 7–9 days post-injection as the critical period for tumor neovascularization, which was initiated from the innate liver vessels via vessel cooption and extended by vascular mimicry and thereof growth of CD34^+^cells. In samples with increasing-sized liver metastases, the infiltrated Ly6C^+^ CD11b^+^ F4/80^−^ monocytes steadily gained the expression of F4/80, a Kupffer cell marker, before transformed into Ly6C^−^ CD11b^int^ F4/80^+^ cells, which, the same phenotype was also adapted by Ly6C^−^ CD11b^−^ F4/80^+^ Kupffer cells. F4/80^+^ TAMs showed proximity to neovascularization and tumor vessels, functionally angiogenic in vivo; and greatly promoted the activation of a few key angiogenic markers such as VEGFA, Ki67, etc. in endothelial cells in vitro. Depletion of macrophages or diversion of macrophage polarization during neovascularization impeded tumor growth and vascularization and resulted in greatly reduced F4/80^+^ TAMs, yet increased CD11b^+^ cells due to inhibition of TAM differentiation. In summary, our results showed dynamic and spatial–temporal F4/80^+^ TAM transformation within the tumor microenvironment and strengthened its role as perivascular and angiogenic TAMs in CRLM.

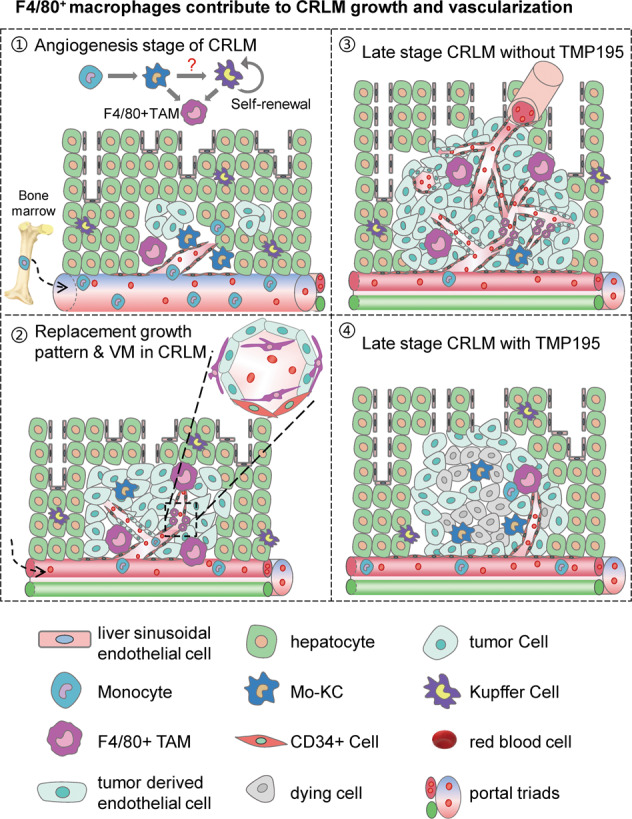

## Introduction

Approximately half of the patients with colorectal cancer (CRC) develop colorectal liver metastases (CRLM), which result in poor prognosis and high mortality [1, 2]. The liver is unique among organs in that it receives blood with differing compositions via both systemic circulation and hepatic portal circulation, which allow the liver cells to perform vital digestive and metabolic functions [2, 3]. The metastatic CRC cells mostly enter the liver via the portal vein; their interactions with the tumor microenvironment play a vital role throughout the disease [3, 4]. Thus, the unique hepatic architecture and immune-tolerant and/or -permissive microenvironment greatly contribute to the tropism for metastatic tumor cells to seed and form CRLM.

CRLM formation can be subdivided into four major stages including a microvascular stage of cancer cell intravasation and arrest in sinusoidal vessels; a pre-angiogenesis stage and an angiogenic stage to build up tumor vasculatures; and a growth stage in which CRLM expands rapidly to form big liver metastases [1, 5]. Among these, the angiogenic stage is an extremely pivotal time to aid the invasion of a growing tumor upon the cells and surrounding liver tissue and result in a series of histopathological growth patterns (HGPs) of CRLM [6, 7]. Vermeulen et al. described three distinct HGPs [7] including a desmoplastic HGP, where the metastases are separated from the liver structure and create their own supporting stroma. In a pushing HGP, where the liver plates at the interface are compressed but have increased angiogenic activity. As for a replacement HGP, the metastatic cells infiltrate and replace the hepatocytes and hijack the liver architecture by aptly adopting sinusoidal vessels, which is achieved mostly via a non-angiogenic process named ‘vessel co-option’ [8]. These HGP subtypes are mainly featured with varied degrees of tumor vasculature by sprouting angiogenesis and disparities in relevant cytokine profiles and immune infiltration, which suggests that different HGPs may reflect immune heterogeneity in the tumor microenvironment [1, 9, 10]. Traditionally, tumor angiogenesis marks the sprouting of new blood vessels lined with endothelial cells. However, Hendrix et al. found that tumor cells may actively create their own blood-delivering tubes and vessels forming a mini-circulatory system [11]. Other studies showed that not only tumor cells, but also other cells in the tumor environment can form tubular structures, such a phenomenon is termed vasculogenic mimicry (VM), which is directed to the high plasticity of the stromal, tumor microenvironment, tumor cells, and cross-talks between tumor and the immune system [12–14].

Kupffer cells, the liver-resident macrophages usually lie in hepatic sinusoids wherein serve as the first line of defense against pathogens entering from the portal and/or arterial circulation and play a role in monitoring hepatic metastases derived from blood-borne tumor cells [15, 16]. However, the surveillance system seems limited in capacity [17, 18]. Until the CRLM reaches the pre-angiogenesis stage, it may have disturbed the sinusoidal vessels and blood flow dynamics and resulted in an ischemic-hypoxic situation, which may incite Kupffer cells to secrete cytokines and chemokines and differentiate into tumor-associated macrophages (TAMs). CRLM progression in time recruits myeloid cells to the liver, among which, monocytes are infiltrated in response to pro-inflammatory signaling including TNF, CCL2, CCL9, etc., and turn into TAMs [17, 19]. Macrophages are strongly adapted to their environment and can have different polarization states with distinct antitumor and tumor-promoting entities, termed as M1- or M2-like [19, 20]. In general, M1-like macrophages are pro-inflammatory and exert antitumor effects, whereas M2-like macrophages largely promote tumor growth via the secretion of growth factors such as VEGF, EGF, FGF2, and TGFβ [21]. However, recent studies indicate transitional or mixed phenotypes of these two entities, even within M1-, or M2-like, there is substantial heterogeneity. For example, M2-like macrophages were further subdivided into M2a, M2b, and M2c, which exhibited distinct cell markers and functions under varied regulatory factors [15, 20]. Both Kupffer cell- and monocyte/macrophage-derived TAMs can promote tumor angiogenesis via secretion of growth factors, inflammatory cytokines, extracellular matrix-degrading proteases to support neovascularization and growth of tumor vessels [9, 17, 22]; and thus, function to promote CRLM progression.

Kupffer cells constitute up to 10% of all liver cells and are constantly replenished via self-renewal in the liver to maintain homeostasis [15, 18]. Under pathological conditions, bone marrow-derived monocyte/macrophages are recruited to the liver on demand, they might act as a backup supplement to Kupffer cells [23]. A recent study elucidated that the recruited monocytes could fully differentiate into Kupffer cells in the liver upon conditional depletion of Kupffer cells in transgenic mice [24]. Under CRLM, understanding how these two sources of macrophages route to tumor-promoting TAMs can provide important clues for effective therapy. In this study, with a C57BL/6 mouse model of CRLM, we showed dynamic growth of CRLM and the formation of tumor vascularization in vivo. Moreover, we delineated the differentiation of F4/80+ TAMs from Kupffer cells and myeloid infiltration of monocytes/macrophages within the tumor microenvironment. F4/80+ TAMs play an essential role in tumor vascularization and can be employed as therapeutic targets in CRLM.

## Results

### Seven to nine days post-injection is the critical period for growth of small-sized liver metastases

**Fig. 1:**
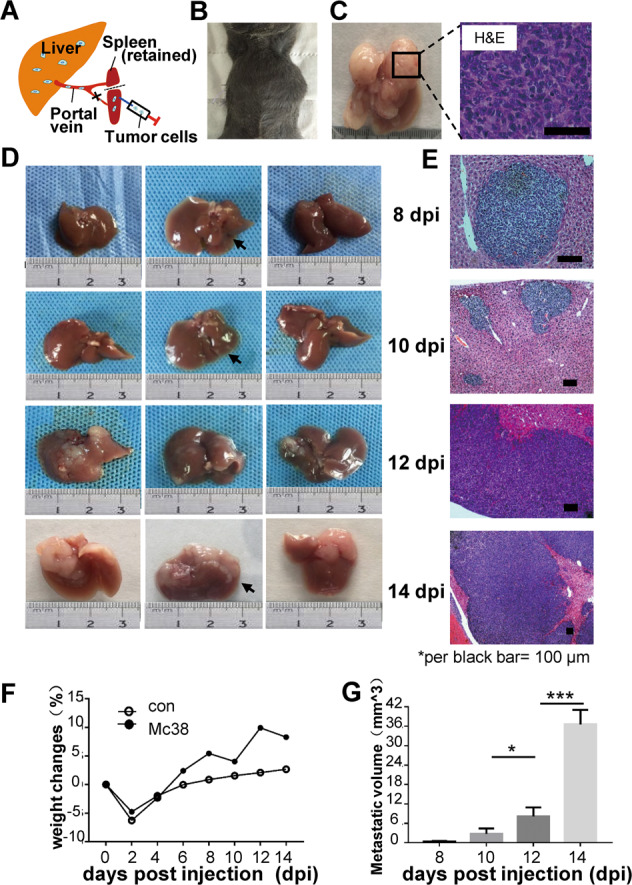
The mouse liver metastasis model and its dynamic disease course. **A** The liver metastasis model in C57BL/6 mice. Mc38 colorectal cancer cells were injected via the spleen-portal vein into the liver. **B** At 21 days post injection (dpi), the abdomen of a tumor-bearing mouse showed large tumor bumps. **C** H&E staining showed huge and non-necrotic liver metastases. Up to 14 dpi, the liver metastases appeared as small to large-sized tumors on the liver surface; arrows pointed to liver metastases (**D**); and under the microscope by H&E staining (**E**). **F** The weight changes in the control and Mc38-injected mice during disease (weight changes were shown in the mean value of 4 mice per group). **G** Metastases volumes in the liver were calculated by measuring the tumor sizes in H&E staining during 8–14 dpi (*N* = 3, with 3–5 fields imaged and quantified). *p < 0.05; ***p < 0.001.

The mouse CRLM model mimicked the late-stage of colorectal liver metastasis in patients, during which the metastatic CRC cells entered the liver via spleen-portal veins and gradually built-up metastases. The mice formed similar liver metastases at 14 dpi (days post-injection), yet most mice suffered from huge liver metastases and cachexia at 21 dpi (Fig. 1A–C). The metastatic niches of only the needle tip size oddly appeared on the liver surface at 8 dpi, with ~1 mm^3^ as calculated according to the correlated H&E staining (Fig. 1D, E). At 10 dpi, the small tumor pieces grew into the sizes of 2–3 mm^3^; whereas at 12 dpi around 8 mm^3^ of metastatic nodules arose on the liver surface, some of them showed bean-shaped tumors. At 14 dpi, large metastases protruded from the liver (Fig. 1D–G). Such a dynamic disease course allowed us to investigate the disease at different stages with the varied tumor microenvironment. Compared to the control mice, changes in the body weight percentages in Mc38-injected mice showed an accelerated growth of the metastases during 10–14 dpi; notably, a relative body weight loss was observed at 8–10 dpi, when it could be assumed as a pivotal transition from pre-angiogenesis to angiogenesis stages to support further CRLM growth (Fig. 1F–G). Together, these results showed that small tumor blocks could be established around 7–9 dpi.

### Replacement growth pattern of the CRLM incorporates liver vessels and vasculogenic mimicry to construct the tumor vasculature

**Fig. 2:**
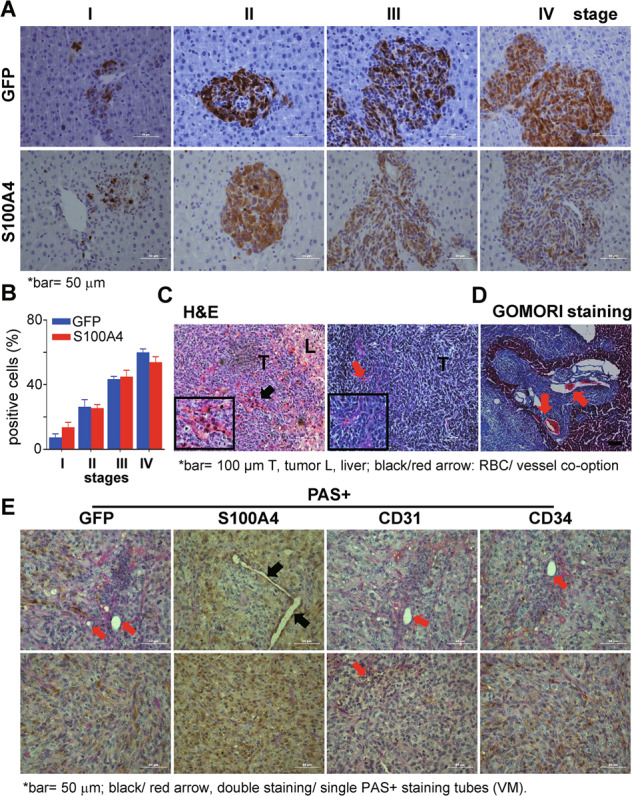
The replacement growth pattern and vascular mimicry (VM) in CRLM. Immunohistochemistry (IHC) with anti-GFP or anti-S100A4 antibodies tracing of Mc38 cells in varied stages showed a replacement growth pattern of CRLM (**A**) and positive staining quantification (**B**)**. C** H&E staining showed remaining hepatic tissues and retained red blood cells within the liver metastases (the red arrow pointed to red blood cells and black to vessel co-option). **D** GOMORI staining showed the tumor–liver interface was smoothly interacted indicating a replacement growth pattern of the liver metastases. **E** Serial IHC sections on the respective double staining with anti-CD34, -CD31, -S100A4, or -GFP antibodies, and Periodic acid-Schiff (PAS) showed CD34^−^ PAS^+^ or CD31^−^PAS^+^ vasculogenic mimicry (VM) tubes (red arrows) and S100A4^+^ PAS^+^ VM (black arrows) within CRLMs.

We used GFP^+^ Mc38 cells to grow CRLM. IHC-staining on the metastasis-associated protein S100 calcium-binding protein A4 (S100A4) showed striking similarity with that of GFP^+^ staining on varied CRLM (Fig. 2A, B), from which we observed a replacement growth pattern. Mc38 cells initially colonized near the hepatic sinusoids or portal veins and co-opted the sinusoidal blood vessels in a stepwise manner (Fig. 2A–C). At 14 dpi, the interface between the tumor and the surrounding liver parenchyma was poorly defined, indicating tumor cells and hepatocytes had intimate cell–cell contact without causing inflammation or fibrosis. H&E showed that some of the liver plates were enclosed at the centers of these tumors, in which the remaining liver sinusoid structures contained well-perfused red blood cells (Figs. 1E, 2C); GOMORI staining manifesting the interface of tumor–liver tissues also showed tumors with nearby portal tracts and co-opted for internal liver vessels (Fig. 2D). Furthermore, GO enrichment analysis with RNA-Sequencing data of varied grade of tumor-centric samples indicated that the most significant events during CRLM progression to the late stages, from medium tumor-sized CRLM (Mt) to high tumor-volume CRLM (Ht) matched with the liver function decline, as most liver metabolism-related genes were significantly down-regulated (Supplementary Fig. S1A the blue lettered GO terms).

We next checked endothelial markers CD34, CD31, and CD146 expression with IHC in vivo. CD34 expression is mostly associated with tumor neovascularization or budding endothelial cells [25], whereas periodic acid Schiff (PAS)^+^ components represent polysaccharides as the intact basement in each tissue. Thus, double staining of CD34^+^ PAS^+^ or CD31^+^ PAS^+^ vessels may represent sprouts of angiogenesis or blood vessels, whereas CD34^−^ PAS^+^ or CD31^−^PAS^+^ tubes showed a pattern of vascularization as non-angiogenesis, named vasculogenic mimicry (VM), in which the tumor cells or non-endothelial cells formed vessels [14, 26]. We observed tubular or circled filaments of single PAS^+^ structures within big metastases, as well as S100A4^+^PAS^+^ tubes (Fig. 2E); Moreover, non-necrotic huge metastases implied well-functioning vessels with continuous blood and gas supply (Fig. 2C–E). We next applied IHC serial section analysis with anti-CD34, -CD31, CD146, or -GFP antibodies, respectively, revealing relations of tumor or blood vessels and GFP^+^ Mc38 cells on varied-sized metastases. We found that, while GFP^+^ Mc38 cells just began to colonize (Supplementary Fig. S1B, S1C); some CD34^+^ staining showed even before tumor cells arrived (Supplementary Fig. S1B and S1C, the 2nd columns: CD34^+^ vs. GFP^+^ staining); later an intensive CD34^+^ staining almost overlapped with that of GFP^+^ on bigger metastases. CD34^+^ staining showed along the walls of liver vessels in which metastases fitted indicating that Mc38 cells co-opted the liver for their colonizing and forming CRLM (Supplementary Fig. S1B, S1C). In contrast, the mature blood vessel endothelial marker CD31^+^ and endothelial adhesion marker CD146 were sparsely shown within CRLM or outside. Therefore, CD34^+^ staining may well represent rapidly budding tumor vessels within CRLM and was used for later analysis (Supplementary Fig. S1B, S1C).

### Dynamics of F4/80^+^ macrophage transformation within CRLM and its correlation with tumor vascularization in vitro and in vivo

**Fig. 3:**
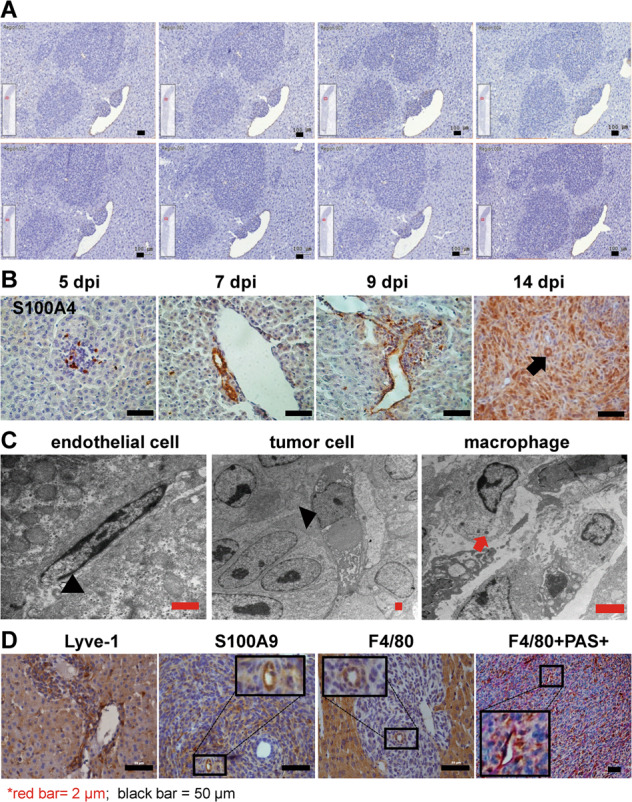
F4/80^+^ macrophages were highly linked to CRLM-adopted liver vessel co-option and VM tubes on tumor vascularization. **A** IHC with an anti-CD34 antibody staining on serial tissue sections of CRLM at 14 dpi displayed 3-D versions of the CD34^+^ tumor mass grown along an innate liver vessel (the elliptical white space). **B** IHC staining on S100A4^+^ cells showing the tumor cells formed tubular structures in a VM manner during disease progression; the arrow showed circular staining with 14 dpi CRLM. **C** Representative transmission electron microscopy showed endothelium-lining vessels, tumor cell-lined tubular structure as VM; and macrophages in proximity to VM tubes. *The arrowhead showed the flatted endothelial cell (left) or a round tumor cell (middle); the red arrow showed a few lysosomes within a macrophage (right). **D** IHC staining on the liver metastases showed tubular expression patterns on respective macrophage-related markers such as Lyve1, S100A9, and F4/80; F4/80^+^ expression showed proximity to PAS^+^ channels.

The reconstructed IHC serial sections on a 14-dpi CRLM displayed a 3D version of a big tumor trunk protruded from a big liver vessel (Supplementary Figs. S2A, 3A). In varied CRLM stages, S100A4^+^ vessels may indicate typical VM tubes progressed along the liver vessels (Fig. 3B). Further Transmission Electron Microscopy (TEM) confirmed the co-existence of the flat-shaped endothelial cells (Fig. 3C left) and round-shaped tumor cells (Fig. 3C middle) and lysosome-containing macrophages (Fig. 3C right) lining the vascular structures. Additional macrophage-related markers i.e., Lyve-1 or S100A9 expressed inside the tumor chunks exhibiting loop-like patterns; F4/80^+^ staining appeared next to PAS^+^ VM tubes (Fig. 3D).

**Fig. 4:**
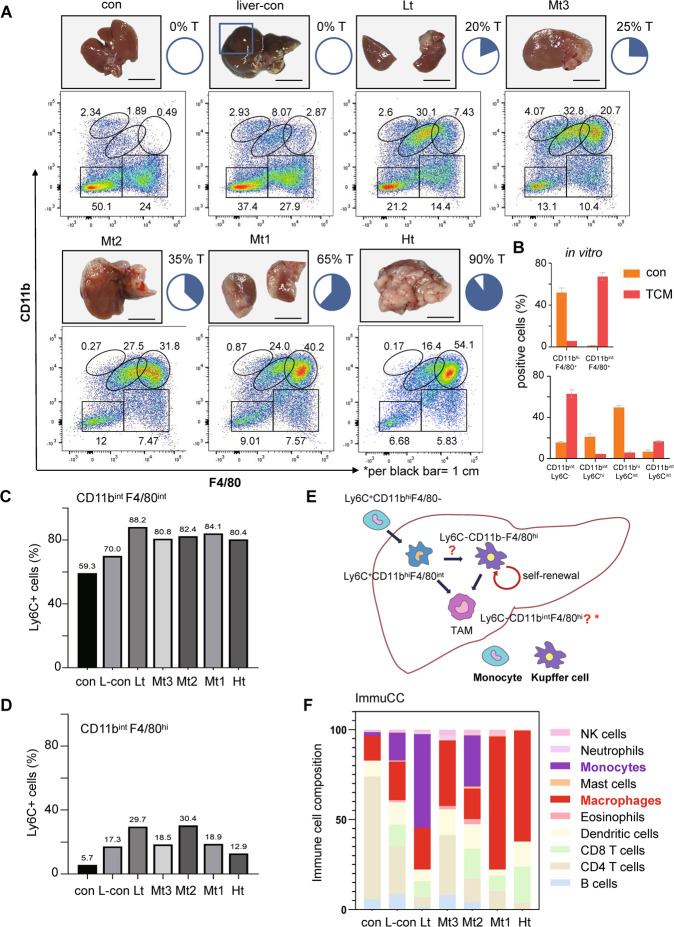
The infiltrated monocytes/macrophages and Kupffer cells transformed into F4/80^+^ TAMs within CRLMs. **A** Tumor-centric samples contained low- (Lt), medium- (Mt3, Mt2, and Mt1), and high- (Ht) tumor-sized liver metastases, the normal liver (con) and the tumor-free liver tissue from the CRLM (liver con), on which flow cytometry analyzed the staining with anti-CD11b and -F4/80 antibodies after CD45^+^ gating (**A**). **B** Co-culture of bone marrow-derived monocytes under Mc38-cell conditioned medium (TCM) for 7 days in vitro confirmed monocyte-transformation into Ly6C^lo^CD11b^int^F4/80^hi^ cells. The relative proportions of Ly6C^+^ cells in CD45^+^ CD11b^int^ F4/80^int^ subsets (**C**) and CD45^+^ CD11b^int^ F4/80^hi^ subsets (**D**) were shown with CRLM samples in (**A**). **E** The schematic diagram showed the routes of F4/80^+^ TAM differentiation from the infiltrated monocytes and Kupffer cells within CRLM. **F** Bioinformatics analysis with ImmuCC algorithm showed relative immune cell content based on the bulk RNA-seq data from the same samples in (**A**); *attention to monocytes (red) and macrophages (purple) in varied samples.

Within CRLM, heterogenous tumor-associated macrophages (TAMs) include myeloid-infiltrated monocyte/macrophage- and Kupffer cell-differentiated TAMs [17]. Using the tumor-centric CRLM samples (Fig. 4A, the tumor biopsy series), we analyzed the dynamic content of infiltrated CD11b^+^ monocytes/macrophages and F4/80^+^ macrophages by parallel flow cytometry and bulk RNA-sequencing. Most F4/80^+^ staining represented Kupffer cells in normal liver and early CRLMs; flow cytometry showed that, during CRLM progression, CD11b^hi^ F4/80^−^ cells entered the liver and became CD11b^int^ F4/80^int^ cells, which further turned into CD11b^int^ F4/80^hi^ cells within large tumors (Fig. 4A, the flow cytometry data). The transformation of Ly6C^hi^CD11b^hi^ cells into Ly6C^lo^ CD11b^int^ F4/80^hi^ cells was confirmed in vitro via co-culture of bone marrow-derived monocytes with Mc38 cell-conditioned medium (TCM) for 7 days (Fig. 4B). Consistently, as more than 80% of CD11b^int^ F4/80^int^ cells were Ly6C^+^ cells (Fig. 4A, C), <30% of Ly6C^+^ cells in CD11b^int^ F4/80^hi^ cells (Fig. 4A, D), we proposed that Ly6C^hi^ CD11b^hi^ monocytes may differentiate into Ly6C^lo/−^ CD11b^int^ F4/80^hi^ macrophages; thus, the increased CD11b^int^ F4/80^hi^ cells were functional TAMs within CRLM (Fig. 4E). Moreover, with parallel RNA-Seq data collected from the same CRLM samples, bioinformatics analysis on the dynamic content of monocyte and macrophages by ImmuCC [27] showed that timing of the monocyte influx and dynamic ratios of monocytes/ macrophages were highly relevant to those by flow cytometry and GO enrichment analysis on DEGs (Fig. 4A and F, Supplementary Fig. S1A) during CRLM growth.

**Fig. 5:**
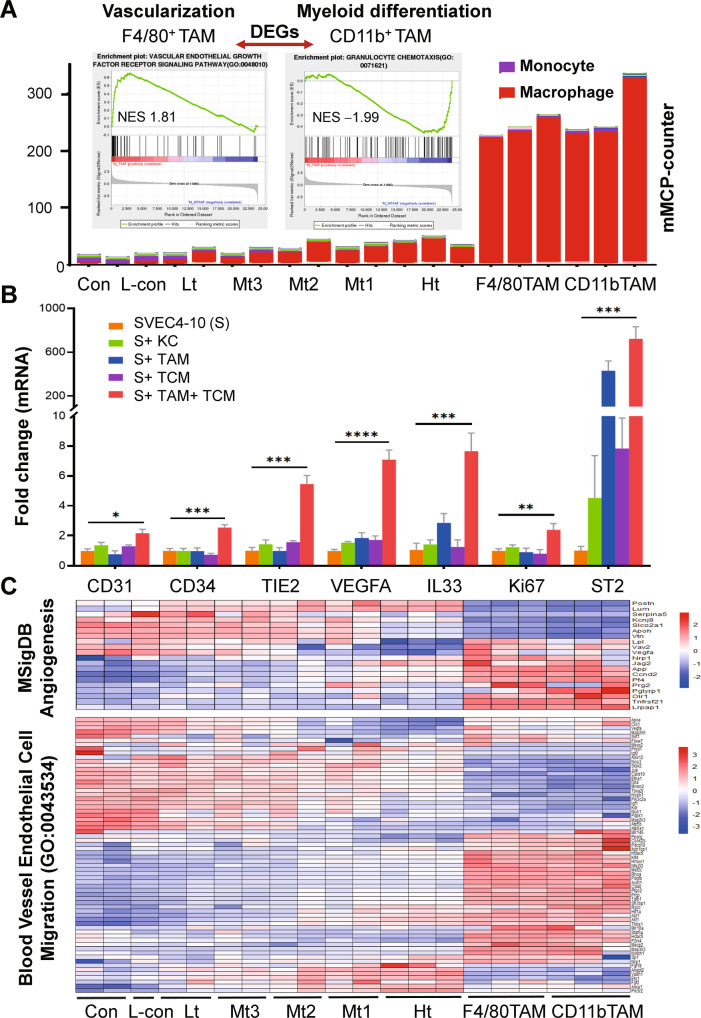
F4/80^+^TAM greatly promoted tumor vascularization in vitro and in vivo. **A** The immune infiltration analysis by mMCP-counter with the bulk RNA-seq data of F4/80^+^ cells and CD11b^+^ cells extracted from CRLM showed mainly macrophages. GSEA analysis showed F4/80^+^TAM was greatly linked to vascularization. **B** Co-culture of ex vivo F4/80^+^TAM from CRLM with the endothelial cells (SVEC4–10, S) under TCM (Mc38 cell conditioned medium) greatly increased the expression of angiogenesis markers in SVEC4–10 cells in vitro by RT-PCR. **C** F4/80^+^TAM and CD11b^+^TAM showed distinct angiogenesis profiles from those in the liver and varied CRLM at the transcriptomic level by bioinformatics analysis. *p < 0.05; **p < 0.01; ***p < 0.001.

We then proved the accumulation of F4/80^+^ macrophages in varied CRLMs on IHC serial sections (Supplementary Fig. S2B). In contrast to normal liver, F4/80^+^ staining during CRLM progression became diffuse-like, light brown, and loop-like patterns along the liver vessel or within CRLM, indicating vital transitions of these cells. Moreover, greatly increased F4/80^+^ cells gathered at the tumor-liver interfaces, became much more at 14 dpi CRLM (Supplementary Fig. S2B). Further IHC data series showed that, throughout the disease esp. 7–9 dpi, F4/80^+^ but not CD11b^+^ staining showed typical tubular-like patterns, signifying the role of F4/80^+^ macrophages in tumor vascularization (Supplementary Fig. S3). To reveal the functions of F4/80^+^ macrophages within CRLM, we extracted F4/80^+^ and CD11b^+^ cells from medium-sized CRLM by magnetic beads (Mt2, Fig. 4A tumor samples) and applied bulk RNA-sequencing analysis. Compared to tissue samples, immune cell infiltration analysis by mMCP-counter (http://134.157.229.105:3838/webMCP/) showed that both cell populations contained mostly macrophages, we thus named after F4/80^+^TAM and CD11b^+^TAM (Fig. 5A). Furthermore, Gene Set Enrichment Analysis (GSEA) analysis on the up-regulated differentially expressed genes (DEGs) indicated significant organogenesis, vascular endothelial growth, etc. on F4/80^+^TAM; yet GSEA on the down-regulated DEGs showed negative regulation of granulocyte chemotaxis, etc. on CD11b^+^ TAM (Fig. 5A inserts). We next proved that in vitro co-culture of the ex vivo F4/80^+^TAMs with the endothelial cells (SVEC4–10) under tumor-cell conditioned media (TCM) greatly stimulated the expression of a few key angiogenic markers including CD31, CD34, TIE2, VEGFA, Ki67, etc. (Fig. 5B). Moreover, we showed that both cell populations, overlapping to a large extent, showed a distinct spectrum of angiogenesis-related activation from those in the liver or bulk CRLM tissues in vivo (Fig. 5C). Taken together, these data highlighted F4/80^+^ TAMs greatly contributed to CRLM vascularization.

### Clodronate liposome-mediated depletion of macrophages during formation of small-sized liver metastases disrupted tumor neovascularization

We next treated the mice at 7–9 dpi of critical neovascularization with 3 doses of Clodronate liposome® (CL) to deplete phagocytic macrophages [28]. The results showed that, at 14 dpi, whereas some of the treated mice showed minor tumors, other treated mice still developed big-sized CRLM indicating treatment resistance (Supplementary Fig. S4A–C). H&E results showed hollow tumors with large necrotic areas in the mice with effective treatment, in contrast to the huge and non-necrotic tumors in the control and treatment-resistant mice (Supplementary Fig. S4D). In IHC data, whereas F4/80^+^ staining was strong within the tumors in the control (Supplementary Fig. S5A the upper channel) and treatment-resistant mice (CL-NS, Supplementary Fig. S5A the bottom channel), the staining was only minimal in effectively CL-treated mice (Supplementary Fig. S5A the middle channels), whose tumor also showed greatly reduced CD146^+^ or CD34^+^ staining (Supplementary Fig. S5A, B). These results showed that effective macrophage depletion at the critical period of tumor formation impeded tumor growth via poor vascularization within CRLM. Further, IF data also showed respective expression on CD68, S100A9, or CD206, which was an M2-like marker, was greatly decreased in CL-treated liver (Supplementary Fig. S5C, D).

### CD11b^int^ F4/80^hi^ TAMs contribute to tumor vascularization within CRLM

**Fig. 6:**
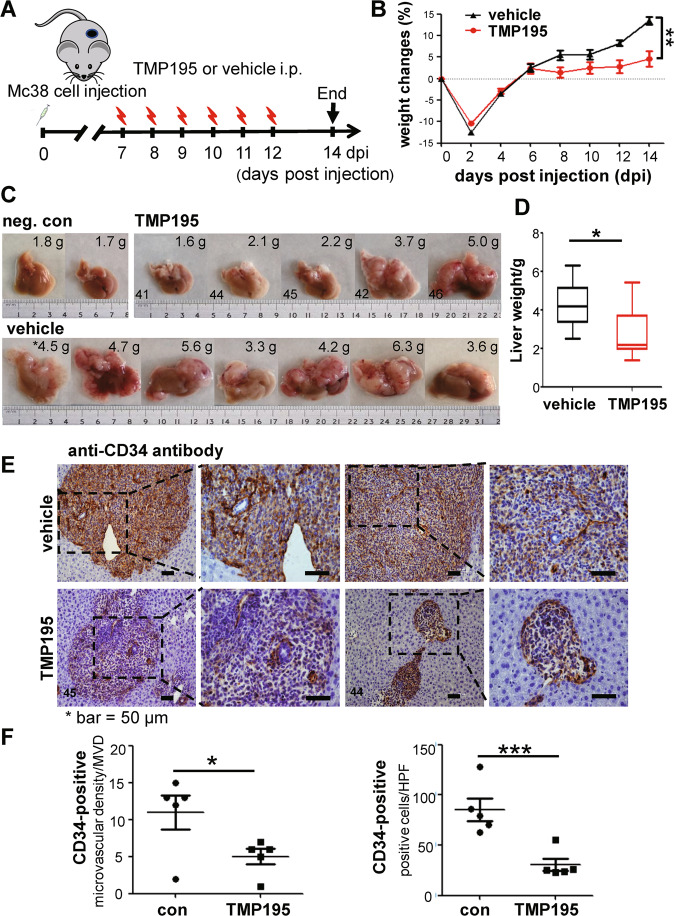
TMP195 treatment greatly inhibited CRLM growth and vascularization. **A** The treatment regimen with TMP195. **B** The weight changes of mice in vehicle and TMP195 groups during 0–14 dpi. **C** The liver and CRLM samples at 14 dpi with/out TMP195 treatment. **D** The liver weights with/out TMP195 treatment at 14 dpi. **E** IHC with anti-CD34 antibodies showed greatly disturbed neovascularization within CRLM in TMP195-treated liver biopsies; *the numbers in the lower left corner of the samples were in relation to the livers as shown (**C**). The treatment significantly decreased CD34^+^ microvascular density (MVD) and the number of CD34^+^-positive cells per HPF (**F**). MVDs were counted in the most active areas of neovascularization per 200× field according to the previous method. *p < 0.05; ***p < 0.001.

We next treated mice with a Class IIa Histone deacetylase (HDAC) inhibitor TMP195 which acted on macrophage polarization towards an M1-like phenotype in vivo [29]. Our results verified that the treatment given during 7–12 dpi greatly disrupted tumor vascularization and inhibited CRLM growth (Figs. 1C and 6A). At 14 dpi, whereas all control and 2 of 5 treated mice showed big metastases, 3 of 5 treated mice significantly less suffered from the disease (Fig. 6B–D). IHC data showed that the treatment greatly undermined CD34^+^ staining showing a hollow appearance within CRLM. In contrast, CD34^+^ expression in the control mice showed greatly more intensive staining with much more rigid wire-like structures (Fig. 6E, F). The treatment also resulted in greatly decreased expression of M2-like marker CD206 yet increased M1 marker iNOS indicating altered macrophage polarization; and greatly decreased expression of CD31 and CD146 confirming corrupted tumor vessels in TMP195-treated livers (Supplementary Fig. S6A, B).

**Fig. 7:**
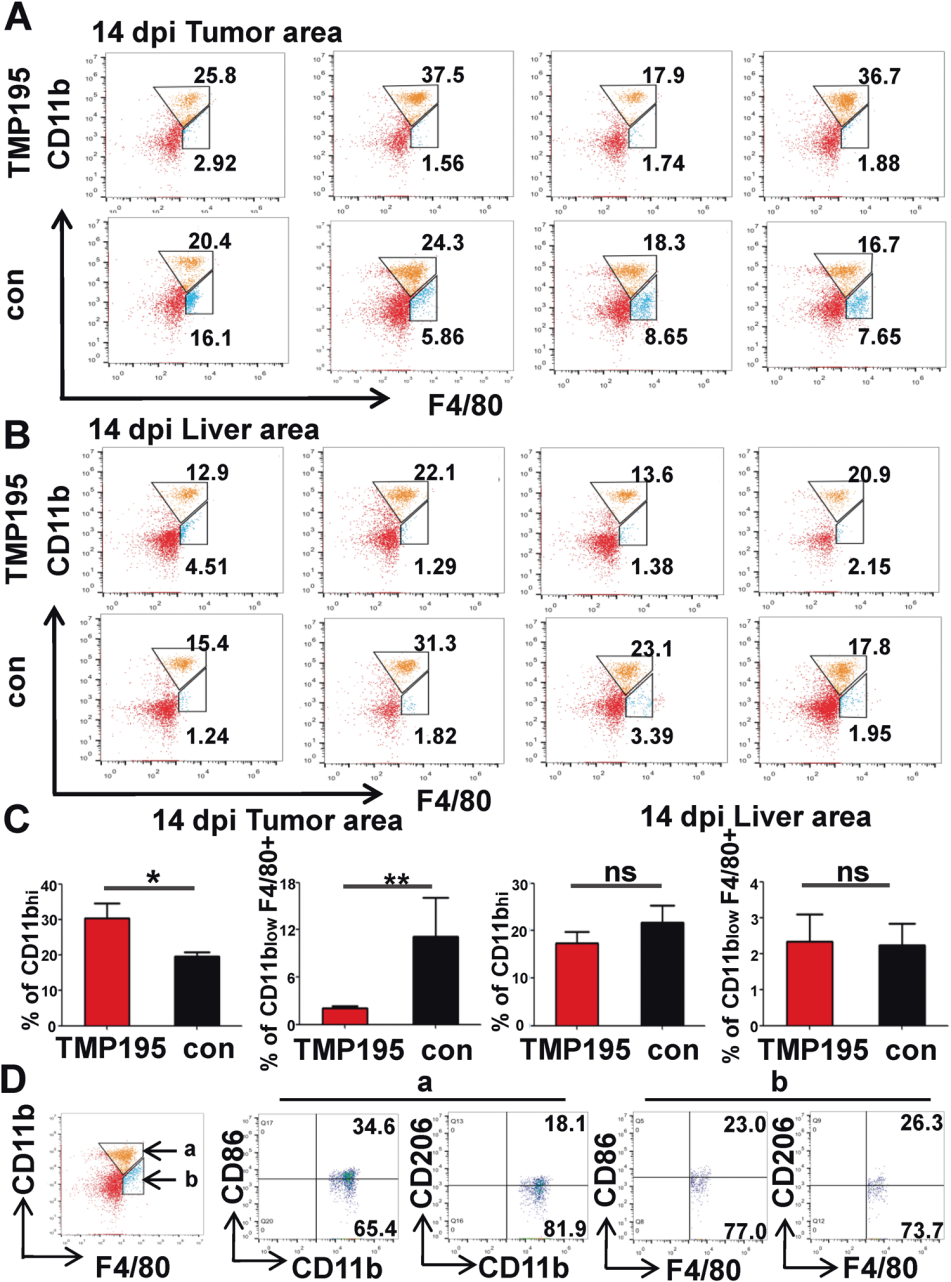
TMP195 treatment depleted CD11b^int^ F4/80^+^ TAMs in late-stage CRLMs. Flow cytometry analysis on 14 dpi liver biopsies of the tumor area (**A**) and liver area (**B**) with/without TMP195 treatment. TMP195 treatment greatly reduced the number of CD11b^int^ F4/80^+^ cells whereas increased that of CD11b^+^ F4/80^−^ cells in the tumors (**A**, **C** left) but not the tumor-free livers (**B**, **C** right) of CRLMs. **D** Further flow cytometry analysis on CD11b^int^ F4/80^+^ (**a**) and CD11b^+^ F4/80^−^ (**b**) cell populations shown in **A** (the lower panel) indicated mixed expression of M1-like type marker CD86 and M2-like marker CD206 in both CD11b^+^ or F4/80^+^ cells. *p < 0.05; **p < 0.01.

Previous data showed that TMP195 treatment did not act on differentiated macrophages but induced the recruitment of CD11b^+^ cells within the tumor microenvironment [29]. In flow cytometry, as compared to the control mice, the treatment significantly reduced CD11b^int^ F4/80^+^ cells but increased CD11b^+^ F4/80^int^ cells within 14 dpi CRLM (Fig. 7A and C); yet these two cell populations kept similar within the liver area between the control and treated mice (Fig. 7B, C). Moreover, CD11b^+^ and F4/80^+^ cells respectively contained mixed M1-like (CD86^+^) and M2-like (CD206^+^) macrophages within CRLM (Fig. 7D). Consistently, the treatment greatly increased CD11b^+^ yet reduced F4/80^+^ expression at 14 dpi CRLM (Supplementary Fig. S7A–D). These results showed that TMP195 treatment resulted in greatly decreased F4/80^+^ TAMs via inhibition of the transformation of CD11b^+^ monocyte/macrophages into F4/80^+^ TAMs within CRLM.

## Discussion

Kupffer cells constitute the largest population of tissue-resident macrophages [15]. These cells generally ensure immune tolerance to avoid undesired immune responses to food antigens, yet they are central to the hepatic response to pathogens and invaders [16]. In our syngeneic mouse CRLM model, survived Mc38 cells in the liver may in time create a tumor microenvironment that favored Kupffer cells to proliferate in situ and/or myeloid monocyte/macrophages to infiltrate, both differentiated into tumor-promoting F4/80^+^ TAMs. Consistently, the replacement growth pattern of CRLM implied that the liver itself was tolerant and supportive of the rapid CRLM growth by actively providing liver vessels at the expense of liver function decline during CRLM progression [7]. The metastatic Mc38 cells made full use of liver vessels and perivascular TAMs; they further adopted VM vessels to match the rapid growth of tumor vasculature. Moreover, we showed that the recruited CD11b^+^ monocyte/macrophages adapted to the local environment by gaining F4/80 expression and transformed into F4/80^+^ TAMs. Our results strengthened the essential roles of Kupffer cells in guiding and contributing to CRLM growth and tumor vessel construction.

Relevant studies show that CRLM growth and liver regeneration shared a few common features regarding tissue environment such as growth factors and cytokines, hemodynamic changes, extracellular remodeling, and angiogenesis [30, 31]. Similarly, the replacement growth pattern of CRLM in our animal model also indicated a permissive liver environment for rapid tumor growth, where liver non-parenchyma cells including Kupffer cells may secret growth factors and cytokines to promote tumor vascularization [31]. However, bigger-sized metastases are mainly fed by the hepatic artery [5]. We showed that neovascularization of CRLM was mostly initiated from an existing liver vessel via vessel co-option; and its rapid growth adopted a replacement growth pattern, where the liver parenchyma contacted the tumor smoothly at the interface [7]. Our IHC showed that CD34^+^ staining dynamics well matched the process of tumor vascularization during CRLM progression, whereas the positive staining of other endothelial cell markers, i.e., CD146 or CD31 did not. The expression of CD34 marks highly functional endothelial progenitor cells in bone marrow [32] and angiogenic tip cells in neovascularization, vessel formation, or lateral branch establishment [33]. Thus, the rapid tumor vasculature construction well adopted CD34^+^ budding endothelial cells leading to swift extension of the existing liver vessels into and around the growing tumor [30]. Moreover, we showed the compensatory endothelial-free tubules with single positive PAS staining indication of vascular mimicry (VM) mechanisms of either tumor cells or F4/80^+^ TAMs, thereby providing an alternative perfusion pathway for CRLM progression [13, 14].

Macrophages are capable of sensing the tissue environment, responding to the needs, and actively maintaining organ function [34]. In early human embryos, tissue macrophages promote tissue regeneration prior to the development of blood vessels [35]. Studies showed macrophages in the perivascular (PV) niche of tumors regulate tumor angiogenesis via direct contact with endothelial cells or pericytes and can form primitive, non-endothelial VM channels in tumor models in vivo [22, 36]. We confirmed F4/80^+^ TAMs as PV-TAMs by spatial and temporal measurement and as unique angiogenic promotors on the endothelial cells in vivo and in vitro. Intensive accumulation of F4/80^+^ macrophages in situ indicating that these TAMs may be highly linked to Kupffer cells and function [37]. Recent data indicated that recruited monocytes, monocyte-derived and resident macrophages may all proliferate in situ to control relevant cell numbers within the tissue [35]; our data on varied stages of CRLM showed that the recruited macrophages may gain F4/80 expression within CRLM, replenishing the pool of Kupffer cells on turning into tumor-promoting TAMs [24, 37]. These facts implied that certain mechanisms may be necessary to unify the recruited monocyte/macrophages to Kupffer cell properties, and the total number of macrophages to coordinate the process of CRLM growth and/or tissue regeneration [30, 34]. We further proved that such a program of monocyte/macrophage diversion to Kupffer-like cells was essential to CRLM progression. Consistent with the diverting effect on macrophage polarization in a breast cancer model in vivo [29], we showed that the treatment of TMP195, a selective class IIa histone deacetylase (HDAC) inhibitor, resulted in an increased number of CD11b^+^ macrophages within CRLM, as well as decreased PV F4/80^+^ TAMs and tumor vessel corruption. Furthermore, effective Kupffer cell depletion with Clodronate liposome (CL) resulted in greatly decreased CRLM growth. Together, these data reflect systemic support and local permission on CRLM growth. Nonetheless, however, recent studies show that, in addition to PV TAMs, neutrophils were also proangiogenic [38, 39] and may have supported metastatic seeding [40, 41] in certain tumor models. We also observed infiltration of myeloid dendritic cells, and CD4^+^ and CD8^+^ T cells into the liver during CRLM progression. Further elucidation of the mechanisms of differential myeloid infiltrations is highly warranted.

In summary, the tumor microenvironment TAMs were derived from macrophage transformation in situ, determined by cells in the local liver microenvironment, and dynamically supplemented by systemic myeloid cells. As a typical example, the CRLM growth is the result of cooperation between local organs and integrated body environment and purposes. How to break the integrated network and reverse the determined tumor growth requires an in-depth understanding of its operating mechanisms and vital points in vivo, which has been partially explained by our data. However, our presented results may only reflect model-specific dynamics and spatial–temporal changes, as liver metastases may grow randomly among varied liver lobules and lobes [1]. Nonetheless, our results imply that a profound understanding of dynamic mechanisms of the tumor microenvironment in disease models may accelerate target discovery that just reverses tumor-promoting entities and effective combination therapies for patients with CRLM.

## Materials and methods

### Cells and animals

Mc38 colorectal cancer cell line was derived from C57BL/6 murine colon adenocarcinoma cells and originally granted from Dr. Shoshana Yakar (New York University) [42] were cultured at 37 °C and 5% CO_2_ in Dulbecco’s modified Eagle’s medium (Hyclone) supplemented with 10% fetal bovine serum (Gibco). The mouse lymph node endothelial cells SVEC4-10 cells (Beijing Zhongyuan Limited, China) were cultured under the same condition. The female C57BL/6 mice, 6–8 weeks (Changsheng Biological Technology Co. Ltd.) were maintained under specific pathogen-free (SPF) conditions. The mouse experiments were approved by the Institutional Animal Care and Use Committees (IACUC) of the Southern University of Science and Technology (No. SUSTC-JY2020093).

### Liver metastasis mouse model in C57BL/6 mice

The mouse liver metastasis model was established via splenic injection of syngeneic Mc38 cells following previous methods as indicated in Fig. 1A [43]. The mice were sacrificed on varied days post injection (dpi) to collect either liver samples or tumor biopsies for further analysis. For hematoxylin and eosin (H&E) staining and tumor sizes in the liver, the mouse body weights were recorded every second day; tumor diameters (*r*) were analyzed by the magnification of relevant H&E-staining images, and matching volumes of the liver metastases were calculated by 4*3.14*r^3^/3.

### H&E, immunohistochemical (IHC) and immunofluorescent (IF) analysis

The collected tissues were fixed, and H&E, IHC, and IF assays were performed by standard procedures (Solarbio), with either a primary antibody (Supplementary Table 1) or the antibody dilution buffer as negative controls. The IHC and H &E samples were analyzed with a Nikon DS-Fi1 microscope; A fluorescence microscope (Nikon 80i) was used to analyze IF images. To quantify the marker-positive cells, three independent fields of the sections were calculated. At least 300 total cells and the positive-staining cells were counted, and the data were reported as percent cells positive for the marker.

### Three-dimensional reconstruction of IHC serial sections

The biopsy of 14 dpi CRLM in the IHC serial section include 100 consecutive slides, 25 of which were scanned into images on TissueFAXS Imaging Software (TissueGnostics, China) and then using Imaris software (Bitplane) to reconstruct the serial images into a short 3-D video (Supplementary Fig. 2A).

### Periodic acid-schiff (PAS) staining and Gomori’s Trichome staining

For PAS staining, the slide sections were treated with periodic acid (Solarbio) before being stained with Schiff’s reagent (Solarbio). For Gomori’s Trichome staining, apply warmed Bouin’s fluid, then Hematoxylin and Weigert’s Iron mixture, Gomori Trichrome Stain solution (ScyTek Laboratories), before differentiating with acetic acid solution and allow further dehydration.

### Co-culture experiments in vitro

Bone marrow-derived monocytes were prepared by flushing the mouse femurs and tibias with PBS with 2% fetal calf serum, plated on a Petri dish, and collected unattached cells post 24 h extraction. Co-cultured the cells with/out tumor cell-conditioned medium (TCM, the cell supernatant from cultured Mc38 cells) for 7 days before harvesting the cells for flow cytometry.

F4/80^+^ cells were extracted from the medium-sized CRLM by microbeads and MS column (130-110-443 and 130-042-201, Miltenyi Biotech) and co-cultured with SVEC4-10 cells with/out TCM for 72 h. The endothelial cells were collected after removing F4/80^+^ cells with microbeads and prepared for RT-PCR analysis.

### Tissue sample preparation for flow cytometry and analysis

Liver biopsy samples with/out varied metastases were prepared for flow cytometry and bulk RNA-Sequencing. Briefly, each tissue sample of ~1 cm^3^ (Volume = length*width*height/2) was used. These samples were tumor-centric and included liver metastases with increasing volumes of the tumor, besides the liver tissue to reflect tumor microenvironment at varied stages of the disease, i.e., low-content tumor (Lt), medium-content (Mt3, Mt2, Mt1), and high-content tumor (Ht) samples which contained more than 90% tumor. Two types of control samples including the tumor-free liver tissue of the 12-dpi mouse liver (L-con), and the control liver tissue. Two livers were prepared for each group except L-con (Fig. 4A). Hepatic mononuclear cell suspensions were prepared from these tissue samples. The cells were stained with the fluorochrome-conjugated antibody for 30 min at 4 °C in the dark and resuspended in FACS buffer before being analyzed in BD FACSCanto SORP.

### RNA-Sequencing samples and data analysis with ImmuCC, mMCP-counter, GO enrichment, Heatmap, and GSEA

A set of aliquot samples (about 0.25 cm^3^) as shown in Fig. 4A were used for bulk RNA-Sequencing, Ht1 and Ht3 were made identical among 14 samples (Novogene, www.novogene.com). F4/80^+^ cells and CD11b^+^ cells were selected from medium-sized CRLM (Mt2) with microbeads and MS column (130-110-443, 130-049-601 and 130-042-201, Miltenyi Biotech) before being prepared for RNA-sequencing.

The resulted RNA-sequencing data clean reads >95%, Q30 > 90%, and error rate <0.05%; Square of Pearson correlation coefficient (*R*^2^) between the biological replicates was >0.89; *R*^2^ of the identical samples (Ht-1 and Ht-3) was >0.98. The hierarchical clustering and principal component analysis (PCA) showed that the biological duplicated samples were clustered together but all grouped into con, L-con, Lt, Mt, and Ht accordingly.

The FPKM files were extracted from all RNA-Seq data and uploaded to ImmuCC web server [27], or mMCP-counter (http://134.157.229.105:3838/webMCP/) to estimate the abundance of tumor-infiltrating immune cells. For Gene Ontology (GO) enrichment analysis, we performed a differential gene expression of two adjacent samples using the edgeR R package (3.22.5), padj ≤ 0.05 and |log2 (fold change)| ≥ 1 as the threshold and GO enrichment analysis by the cluster Profiler R package (3.8.1). Biological process (BP) GO terms with corrected *p* value < 0.05 were considered significant. The heatmap plot was generated using the R pheatmap package; the angiogenesis-related gene sets were taken from the MSigDB and Gene ontology (GO) databases. The up-regulated and down-regulated differentially expressed genes (DEGs) between RNA-seq data of F4/80^+^ cells and CD11b^+^ cells were further applied to gene set enrichment analysis (GSEA) analysis.

### Macrophage depletion and modulation of macrophage polarization in vivo

The mouse was randomized before treatment so that each group had animals of similar weight and age; no blinding was done. For depletion of macrophages in vivo, Clodronate liposomes (CL, Liposoma BV) or PBS liposomes (200 μl/mouse) were injected intraperitoneally at 7, 9, and 11 dpi of the CRLM. For modulation of macrophage polarization in vivo, a class IIa Histone deacetylase (HDAC) inhibitor, TMP195 (MedChemExpress), or the solvent DMSO was injected intraperitoneally. The mice were treated with either solvent or TMP195 of 50 mg/kg daily from 7 to 12 dpi before all biopsies were collected at 14 dpi.

### Transmission electron microscopy

The samples of the liver metastases were prepared as 1 mm^3^ samples before embedded in Araldite. The sections were observed under a transmission electron microscope (H-765, Hitachi).

### Statistical analysis

Data were presented as mean ± SD and analyzed with the unpaired, two-tailed Student’s *t*-test. Statistical analysis was performed on the Prism 7.0 software (GraphPad), and *p* < 0.05 was considered significant.

## Supplementary information

A reproducibility checklist

Supplementary Figure 1

Supplementary Figure 1

Supplementary Figure2A

Supplementary Figure 2

Supplementary Figure 3

Supplementary Figure 4

Supplementary Figure 5

Supplementary Figure 6

Supplementary Figure 7

Supplementary Table 1

Supplementary Figures Legends

The supplementary data summary

## Data Availability

All data generated or analyzed during this study are included in this published article and its supplementary information files, further inquiries can be directed to the corresponding author HR. RNA-Sequencing data were deposited at the GEO database: accession number GSE206211.
