# Supplementary figures and images for: Dynamic differentiation of F4/80+ tumor-associated macrophage and its role in tumor vascularization in a syngeneic mouse model of colorectal liver metastasis
Source: Cell Death Dis. 2023 Feb 13;14(2):117. doi: 10.1038/s41419-023-05626-1 (PMC9925731; doi:10.1038/s41419-023-05626-1)

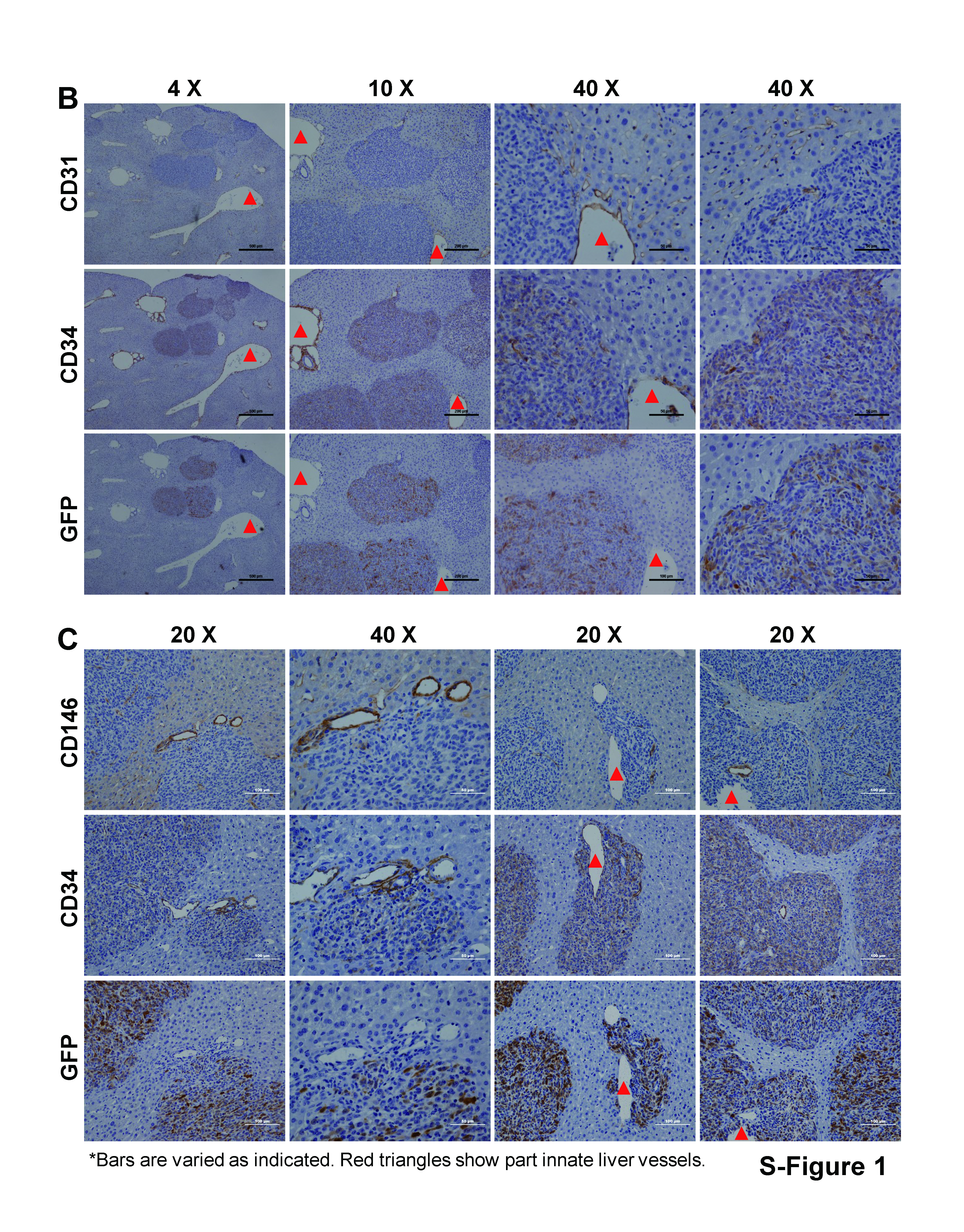

Supplement: Supplementary file 3 — Supplementary Figure 1 [file 41419_2023_5626_MOESM3_ESM.tif]

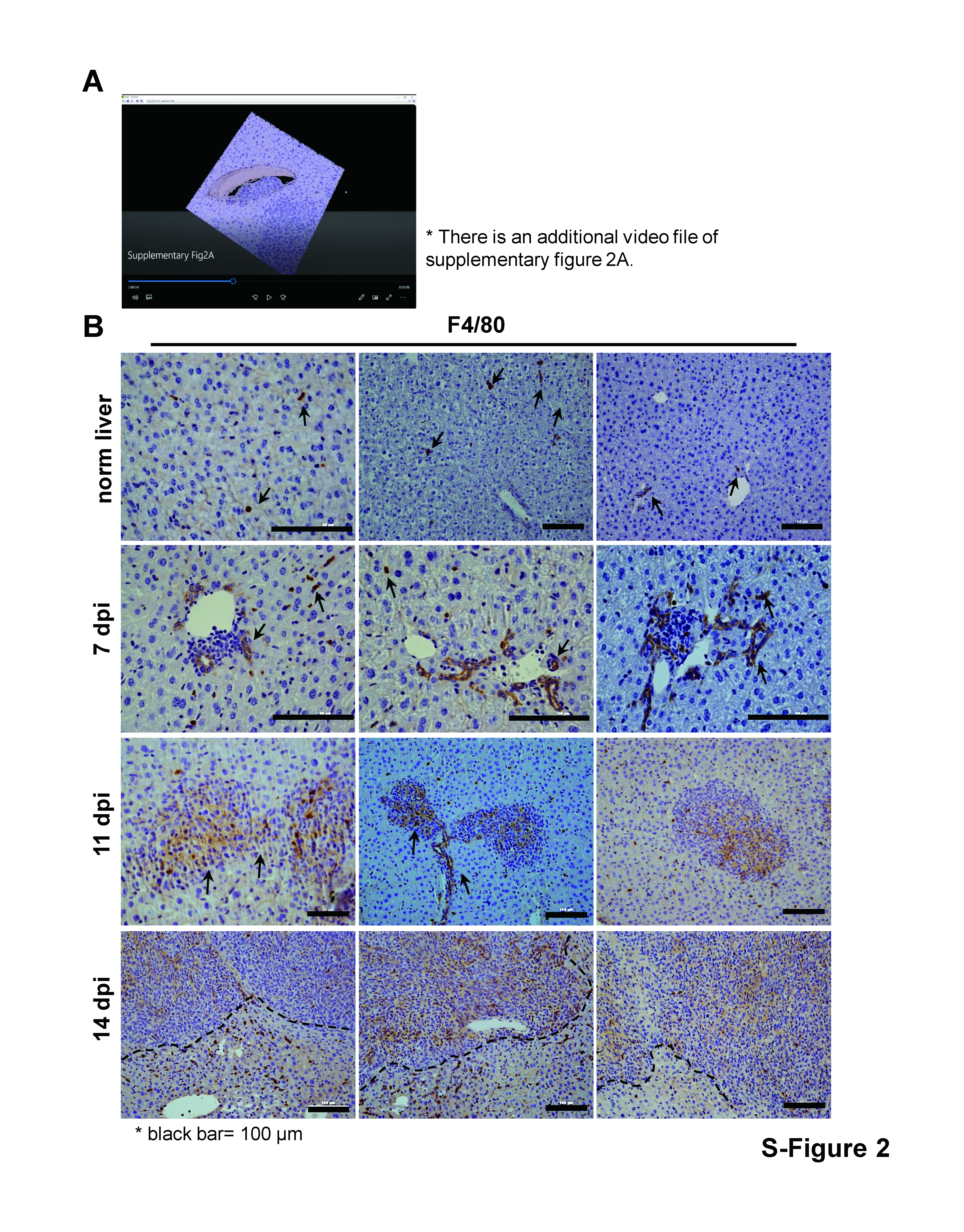

Supplement: Supplementary file 5 — Supplementary Figure 2 [file 41419_2023_5626_MOESM5_ESM.tif]

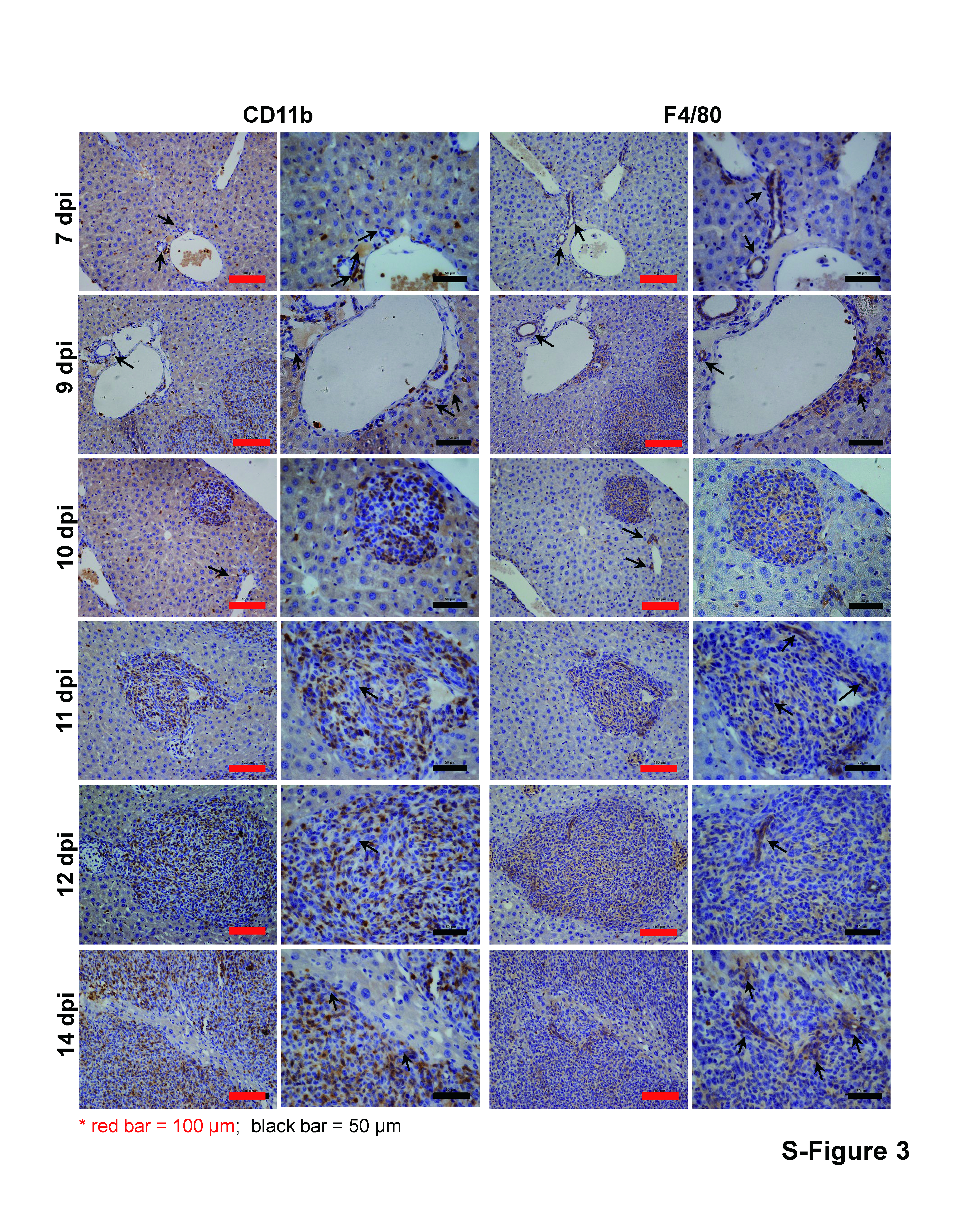

Supplement: Supplementary file 6 — Supplementary Figure 3 [file 41419_2023_5626_MOESM6_ESM.tif]

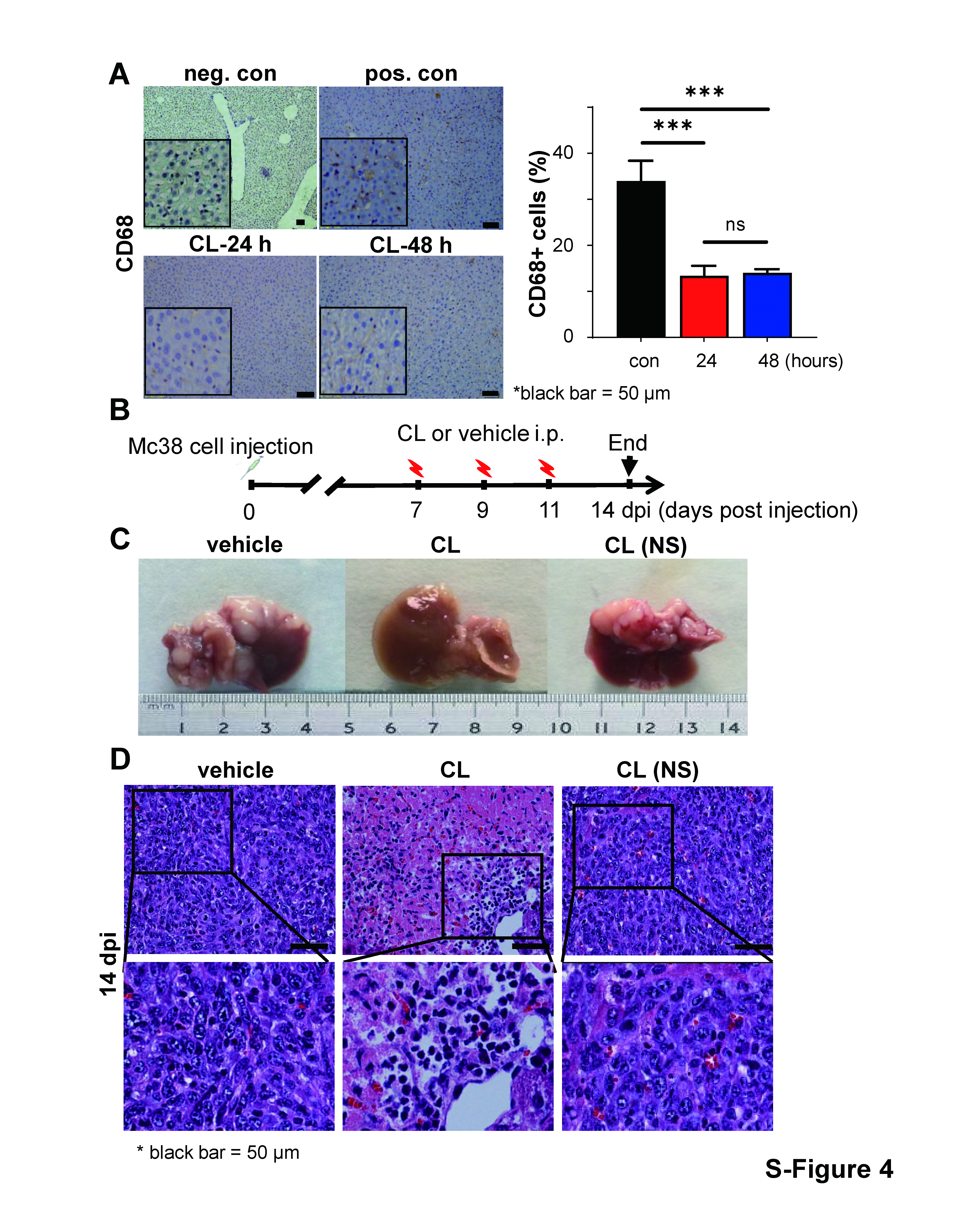

Supplement: Supplementary file 7 — Supplementary Figure 4 [file 41419_2023_5626_MOESM7_ESM.tif]

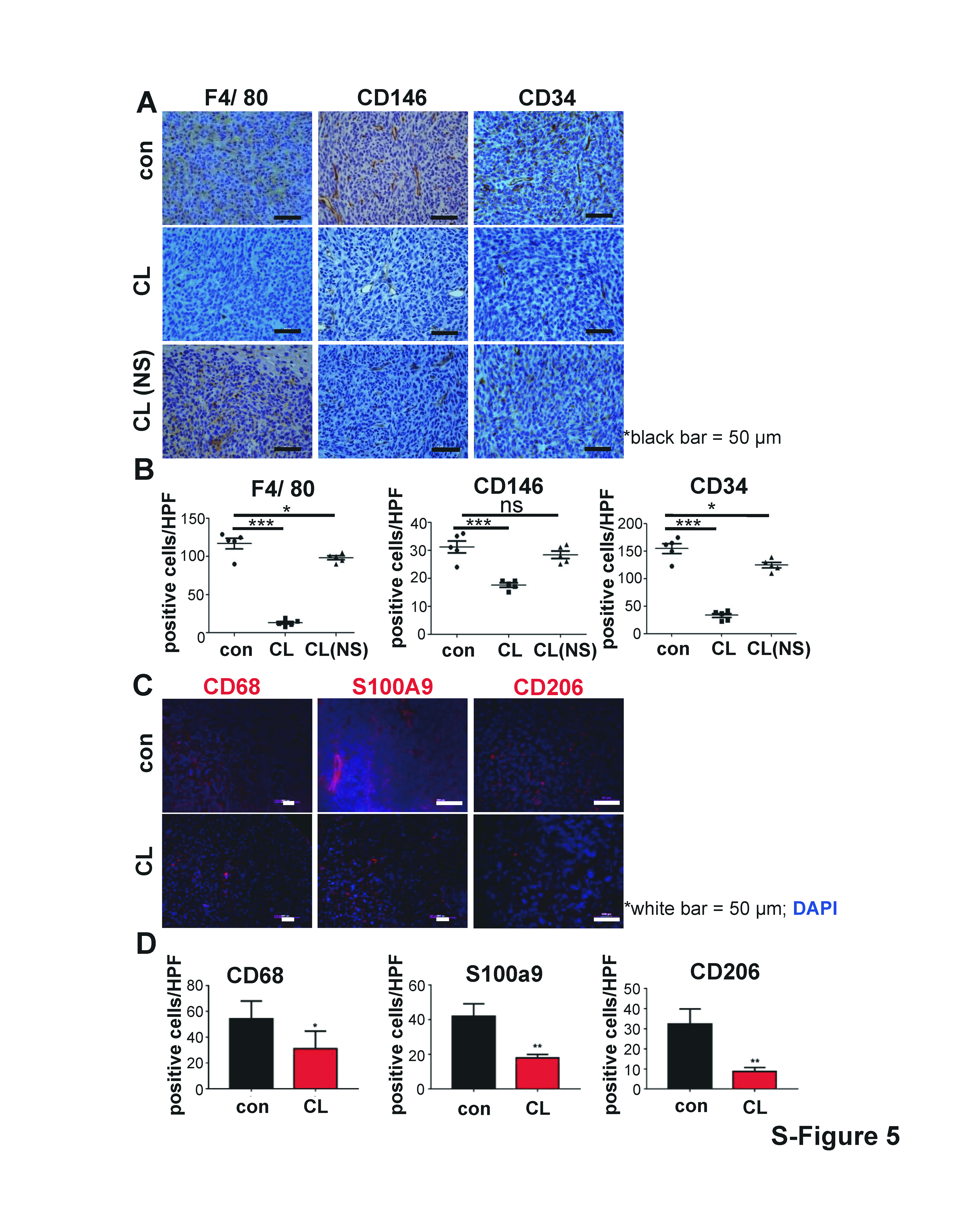

Supplement: Supplementary file 8 — Supplementary Figure 5 [file 41419_2023_5626_MOESM8_ESM.tif]

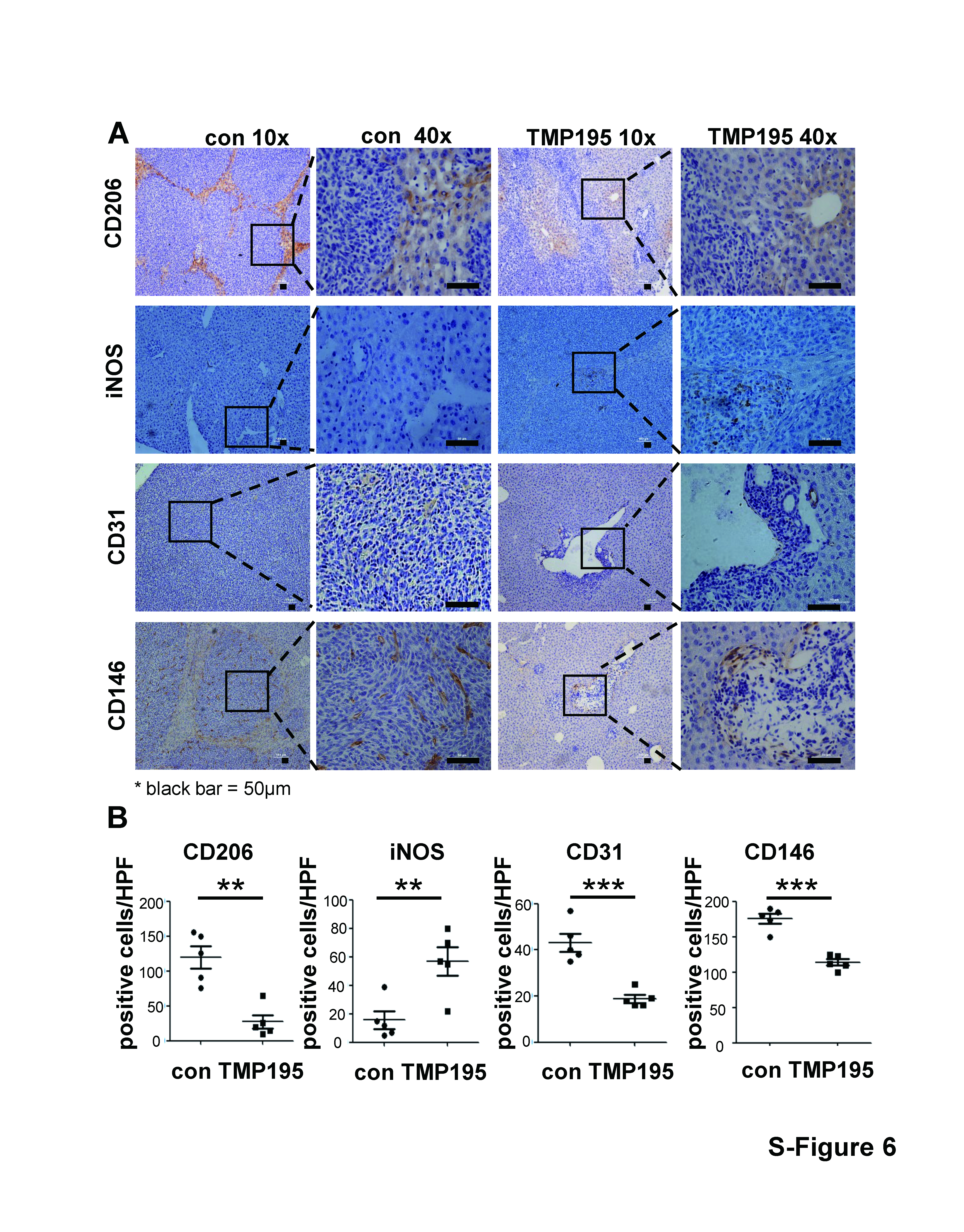

Supplement: Supplementary file 9 — Supplementary Figure 6 [file 41419_2023_5626_MOESM9_ESM.tif]

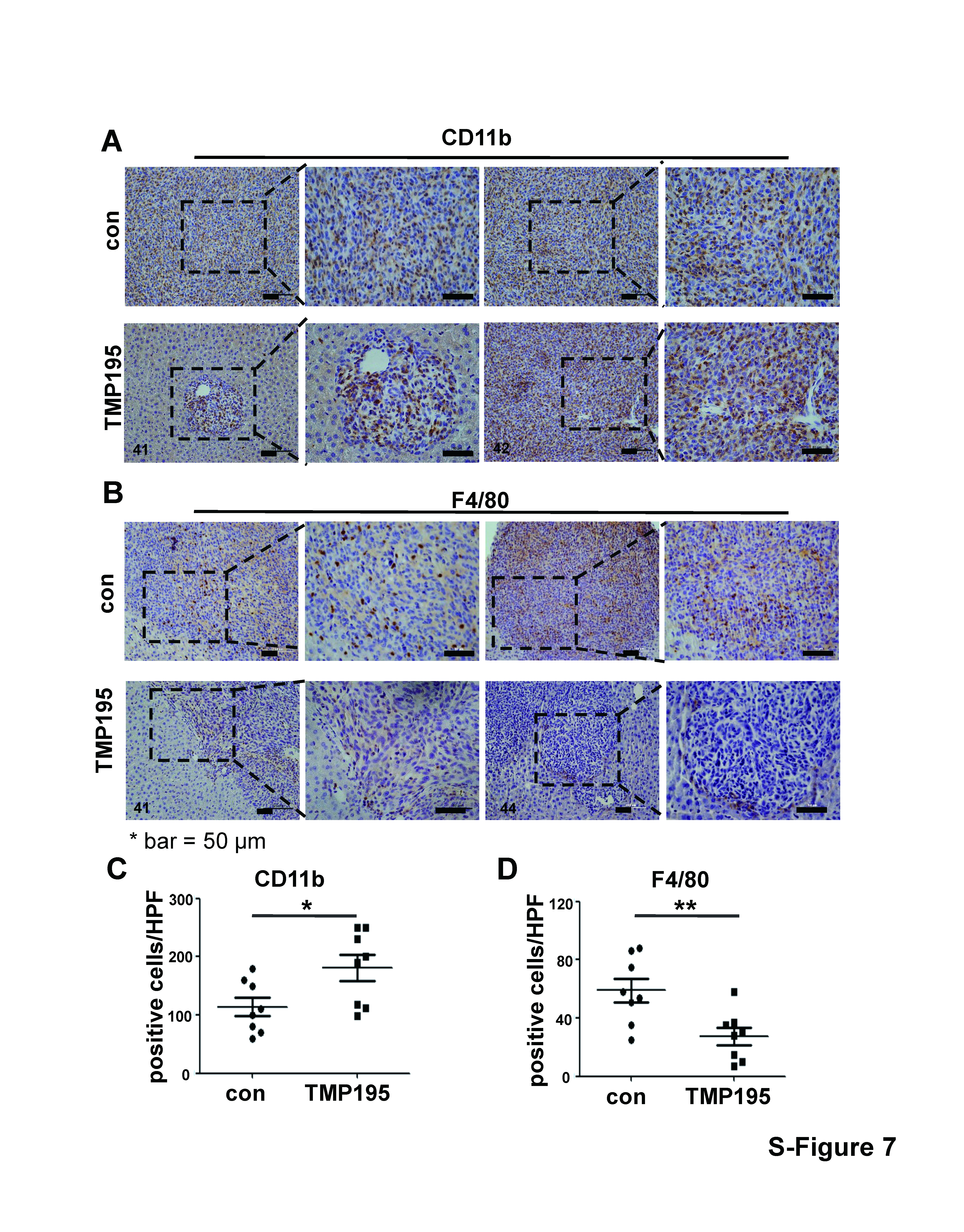

Supplement: Supplementary file 10 — Supplementary Figure 7 [file 41419_2023_5626_MOESM10_ESM.tif]
