# Supplementary material for: Dynamic differentiation of F4/80+ tumor-associated macrophage and its role in tumor vascularization in a syngeneic mouse model of colorectal liver metastasis
Source: Cell Death Dis. 2023 Feb 13;14(2):117. doi: 10.1038/s41419-023-05626-1 (PMC9925731; doi:10.1038/s41419-023-05626-1)
Supplement: Supplementary file 11 — Supplementary Table 1 [file 41419_2023_5626_MOESM11_ESM.docx]

| Antibody | Cat. | Company | Application | Dilution Used |
| --- | --- | --- | --- | --- |
| rabbit anti-GFP | ab183735 | Abcam | IHC | 1:200 |
| rabbit anti-S100a4 | ab197896 | Abcam | IHC | 1:1000 |
| rabbit anti-CD34 | ab81289 | Abcam | IHC/IF | 1:1000 |
| rabbit anti-F4/80 | ab111101 | Abcam | IHC | 1:200 |
| rabbit anti-S100 | ab183979 | Abcam | IHC | 1:1500 |
| mouse anti-S100a9 | ab22506 | Abcam | IHC/IF | 1:1000 |
| rabbit anti-Lyve1 | ab33682 | Abcam | IHC | 1:300 |
| mouse anti-CD68 | ab955 | Abcam | IHC/IF | 1:200 |
| mouse anti-CD206 | ab64693 | Abcam | IHC/IF | 1:2500 |
| rabbit anti-iNOS | ab15323 | Abcam | IHC | 1:100 |
| rabbit anti-CD146 | ab75769 | Abcam | IHC | 1:300 |
| rabbit anti-CD31 | ab28364 | Abcam | IHC | 1:200 |
| rabbit anti-CD11b | ab133357 | Abcam | IHC | 1:4000 |
| anti-mouse CD45-FITC | 103108 | BioLegend | FC | 1:200 |
| anti-human and mouse CD11b-PE | 130-113-235 | Miltenyi | FC | 1:50 |
| anti-mouse/human CD11b-APC/Fire™ 750 | 101262 | BioLegend | FC | 1:100 |
| anti-mouse F4/80-FITC | 130-117-509 | Miltenyi | FC | 1:50 |
| anti-mouse F4/80-Alexa Fluor® 647 | 123122 | BioLegend | FC | 1:200 |
| anti-mouse CD86-Percap | 105026 | BioLegend | FC | 1:100 |
| anti-mouse CD206-APC | 141708 | BioLegend | FC | 1:50 |
| anti-mouse Ly-6C-Brilliant Violet 42™ | 128032 | BioLegend | FC | 1:100 |

**Supplementary Table 1: Antibodies table**

IHC – Immunohistochemical

IF – ImmunoFluorescence

FC – Flow Cytometry
