# Supplementary material for: Dynamic differentiation of F4/80+ tumor-associated macrophage and its role in tumor vascularization in a syngeneic mouse model of colorectal liver metastasis
Source: Cell Death Dis. 2023 Feb 13;14(2):117. doi: 10.1038/s41419-023-05626-1 (PMC9925731; doi:10.1038/s41419-023-05626-1)
Supplement: Supplementary file 12 — Supplementary Figures Legends [file 41419_2023_5626_MOESM12_ESM.docx]

Supplementary Material

# Supplementary Figures Legends

**Supplementary Fig 1.** **CD34^+^ neovascularization played an important role in CRLM vasculature formation and growth. (A)** Functional transitions in CRLM progression by GO enrichment analysis. Bulk RNA-Sequencing data were from aliquots of serial samples as shown in Fig. 4A. Top 6 significant GO terms (Biological Processes, BP) from GO enrichment analysis on the deferentially expressed genes (DEGs) between the neighboring samples were shown (arrows). *The red letters indicated most of the DEGs associated with these GO terms were up-regulated genes, whereas the blue letters involved down-regulated genes. The grey bars indicated the varied adjusted *p* values. The results reflected dynamic liver environment alterations and gradual liver function decline with CRLM growth. (**B**) The IHC serial sections with anti-CD31, -GFP and -CD34 antibodies on varied CRLM. CD34^+^ staining was accompanied with that of GFP^+^ during CRLM growth. CD34^+^ vascular proliferation was initiated from innate liver vessels and appeared even before GFP^+^ tumor cells colonization indicating liver preparation for Mc38 cell colonization and growth. CD31-positive staining was shown along the wall of innate liver vessels but few within the liver metastases. (**C**) The IHC serial sections with anti-CD146, -GFP and -CD34 antibodies on varied CRLM showed GFP^+^ and CD34^+^ staining in relevant to CD146^+^ staining. CD146-positive staining was shown on blood vessels around the tumors and a few within the metastases. *The red triangles indicated the lumen space of part of the innate liver vessels. These data were from repeated IHC experiments at least 3 times.

**Supplemental Fig 2. F4/80 expression was accompanied with dynamic CRLM growth. (A)** The liver metastases grown on a liver vessel in 3-D with an anti-CD34 antibody staining by IHC. Three-dimensional reconstruction on 25 of the serial section micrographs of the 14-dpi CRLM as indicated in Materials and methods. A total of one hundred slides each of 5 µm serial sections were made from a 14- dpi CRLM. **(B)** Dynamic staining of F4/80^+^ macrophages in CRLM. The IHC staining showing F4/80^+^ Kupffer cells in normal liver (the top channel) and dynamic changes of F4/80^+^ macrophages within CRLM. At 7 dpi, F4/80-positive staining accumulated around the liver vessels and showed tubular expression patterns; at 11 dpi, F4/80+ macrophages infiltrated into the liver metastases, some of the staining around the border and showed tubular expression pattern; At 14 dpi, intensive F4/80^+^ staining within liver metastases and around the tumor mass. *Note the staining patterns were different between the tissue within tumor mass showing a light and diffusive brown staining, and the tissue in the liver or outside the tumor as dark brown dotted staining.

**Supplemental Fig 3. Dynamic F4/80+ vs CD11b+ expression within CRLM.** The IHC serial sections with anti-CD11b and anti-F4/80 antibodies on varied stages of CRLM indicated F4/80^+^ not CD11b^+^ staining showed typical tubular-like patterns.

**Supplemental Fig 4. Depletion of macrophages inhibited CRLM growth.** (**A**) IHC on CD68^+^ macrophages and the quantification of macrophage depletion in the liver before and after treatment with Clodronate Liposomes (CL) in mice. (**B**) The CL treatment regimen in CRLM. (**C**) CRLM at 14 dpi with/without CL treatment. *CL (NS) indicated the treatment was resistant and not significant. **(D)** H&E staining showed large necrotic areas in CRLM at 14 dpi in CL-treated liver biopsies as compared with the control and CL(NS). *** *p*< 0.001; ns, not significant.

**Supplemental Fig 5. CL treatment disturbed CRLM vasculature and affected the expression of macrophage-related markers.** (**A**) IHC staining with anti-F4/80, anti-CD146 and anti-CD34 antibodies in CL-treated, control and CL(NS) liver biopsies and respective quantifications of positive staining cells per HPF in (**B**). **p*< 0.05; ****p*< 0.001 (**C**) Immunofluorescent staining with anti-CD68, anti-S100a9, and anti-CD206 antibodies (red fluorescence) in CL-treated and control liver biopsies and respective quantification of positive staining cells per HPF in (**D**). **p*< 0.05; ***p*< 0.01; ****p*< 0.001

**Supplemental Fig 6. TMP195 treatment modulated macrophages towards a M1-like phenotype and disturbed tumor vascularization within CRLM.** (**A**) IHC staining on the M2-like macrophage marker CD206, M1-like marker iNOS, and the endothelial markers CD31 and CD146 in TMP195 treated/untreated CRLM; and the respective quantifications of positive staining cells per HPF in **(B)**. ***p*< 0.01; ****p*< 0.001

**Supplemental Fig 7. TMP195 treatment decreased the expression of F4/80 but increased that of CD11b within CRLM.** (**A**) IHC staining with anti-CD11b antibody showed a significant increase in CD11b^+^ expression in TMP195 treated CRLM as compared to control mice. (**B**) IHC staining with anti-F4/80 antibodies decreased F4/80^+^ expression in TMP195 treated CRLM. Quantification of positive staining cells per HPF on CD11b staining in (**C**) and F4/80 staining in (**D**) in TMP195 treated and untreated CRLM. **p*< 0.05; ***p*< 0.01
