# Supplementary material for: Dynamic differentiation of F4/80+ tumor-associated macrophage and its role in tumor vascularization in a syngeneic mouse model of colorectal liver metastasis
Source: Cell Death Dis. 2023 Feb 13;14(2):117. doi: 10.1038/s41419-023-05626-1 (PMC9925731; doi:10.1038/s41419-023-05626-1)
Supplement: Supplementary file 13 — The supplementary data summary [file 41419_2023_5626_MOESM13_ESM.docx]

The supplementary materials provided further data on the dynamics of F4/80+Kupffers cells，CD11b+monocytes of liver infiltration and tumor vasculatures in the mouse model of colorectal liver metastasis in vivo mainly by serial sections of Immunohistochemistry; data series on the experiments of targeted depletion of macrophages in the model, etc.
